# Socioeconomic Differences in the Effectiveness of the Removal of the “Light” Descriptor on Cigarette Packs: Findings from the International Tobacco Control (ITC) Thailand Survey

**DOI:** 10.3390/ijerph8062170

**Published:** 2011-06-14

**Authors:** Mohammad Siahpush, Ron Borland, Geoffrey T. Fong, Tara Elton-Marshall, Hua-Hie Yong, Charamporn Holumyong

**Affiliations:** 1 Department of Health Promotion, Social and Behavioral Health, College of Public Health, University of Nebraska Medical Center, 986075 Nebraska Medical Center, Omaha, NE 68198, USA; 2 The Cancer Council Victoria, 100 Drummond Street, Carlton, Victoria 3053, Australia; E-Mails: Ron.Borland@cancervic.org.au (R.B.); Hua.Yong@cancervic.org.au (H.-H.Y.); 3 Psychology Department, University of Waterloo, 200 University Avenue West, Waterloo, Ontario N2L3G1, Canada; E-Mail: gfong@uwaterloo.ca; 4 Propel Center for Population Impact, University of Waterloo, 200 University Avenue West, Waterloo, Ontario N2L3G1, Canada; E-Mail: teelton@uwaterloo.ca; 5 1382 Soi Panitchayakan 28, Charansanitwong 13 Rd, Thapra, Bangkokyai, Bangkok, 10600, Thailand; E-Mail: joycharam@gmail.com

**Keywords:** tobacco control policies, socioeconomic position, light cigarettes

## Abstract

Many smokers incorrectly believe that “light” cigarettes are less harmful than regular cigarettes. To address this problem, many countries have banned “light” or “mild” brand descriptors on cigarette packs. Our objective was to assess whether beliefs about “light” cigarettes changed following the 2007 removal of these brand descriptors in Thailand and, if a change occurred, the extent to which it differed by socioeconomic status. Data were from waves 2 (2006), 3 (2008), and 4 (2009) of the International Tobacco Control (ITC) Thailand Survey of adult smokers in Thailand. The results showed that, following the introduction of the ban, there was an overall decline in the two beliefs that “light” cigarettes are less harmful and smoother than regular cigarettes. The decline in the “less harmful” belief was considerably steeper in lower income and education groups. However, there was no evidence that the rate of decline in the “smoother” belief varied by income or education. Removing the “light” brand descriptor from cigarette packs should thus be viewed not only as a means to address the problem of smokers’ incorrect beliefs about “light” cigarettes, but also as a factor that can potentially reduce socioeconomic disparities in smoking-related misconceptions.

## 1. Introduction

Wide and persisting socioeconomic disparities in smoking exist throughout the World [1–8]. Smokers from lower socioeconomic backgrounds have a lower awareness of the harms of smoking, higher levels of nicotine dependence, lower self-efficacy to quit, and are less likely to have an intention to quit [9–11]. While there is a growing literature on the effect of tobacco control policies on smoking-related behaviors, beliefs, and attitudes, less attention has been given to how this effect varies by socioeconomic position. A few studies have shown that increasing the price of cigarettes results in a larger decrease in smoking prevalence among lower income and occupational groups [12–15], while other studies have reported no socioeconomic difference [16]. Anti-smoking mass media campaigns are shown to be equally effective across socioeconomic groups in reducing smoking prevalence [17] and promoting calls to telephone quitlines [18], but to be more effective in lower socioeconomic groups in enhancing cessation rates [19]. One study, however, has reported that mass media campaigns are more effective in promoting quit attempts among higher *versus* lower-educated smokers [20]. More work is needed to identify or develop tobacco control policies that are effective in lower socioeconomic groups and can potentially reduce smoking disparities. The purpose of this article was to assess some of the effects of the 2007 policy of banning “light” descriptors on cigarette packs in Thailand.

Terms such as “light” or “mild” have been used by tobacco companies typically to distinguish cigarettes with low machine-tested yields of tar and nicotine, and weaker taste accompanied by less irritation to the throat and chest [21]. A key engineering feature of “light” cigarettes is filter ventilation. While many smokers believe that “light” cigarettes deliver less tar and are “healthier” or less harmful than regular cigarettes [21,22], there is ample evidence that “light” cigarettes provide no health benefits and actually may cause extra harm by encouraging some smokers, who might otherwise attempt to quit, to continue smoking with the false belief that they are reducing their health risk by smoking “light” cigarettes [23]. The main reason “light” cigarettes do not reduce harm is that lower machine-measured nicotine yields is counteracted by compensatory smoking behaviors, such as taking more and larger puffs, inhaling more deeply, and blocking filter ventilation to increase smoke concentration and regulate nicotine intake [24]. Smokers therefore receive similar amounts of nicotine and tar.

Article 11 of the World Health Organization’s (WHO) Framework Convention on Tobacco Control (FCTC) calls for laws that ensure that tobacco packaging and labeling do not promote tobacco products by any means, such as the use of “light” and “mild” descriptors, that create a false impression of their health hazards. Brazil in 2001 and the European Union (EU) in 2003 were among the first jurisdictions to have prohibited the use of “light” and “mild” descriptors on cigarette packs [25]. In March 2007, Thailand also implemented this policy. All cigarettes that were manufactured in or imported to Thailand prior to this date were exempt from complying with this law for six months. It should be noted that in response to the ban, the tobacco industry has used pack colors to continue to convey that some cigarettes are lighter or of lower tar. The color red denotes regular or full-flavor, the colors blue and gold denote “light” or “mild”, and green denotes “menthol”. The tobacco industry has also used paper thickness of packaging to denote type of cigarettes, with hard packages denoting regular and soft packages denoting “light” or “mild”. Previous research shows that in 2005, 37.9% of smokers in Thailand used “light” cigarettes, and that 43% and 59% of smokers believed that “light” cigarettes are less harmful and smoother, respectively [26]. The aim of this study was to assess changes in beliefs about “Light” cigarettes in Thailand following their removal in 2007 and whether changes in beliefs differed across levels of income and education.

## 2. Methods

### 2.1. Data

The data came from waves 2 (2006), 3 (2008), and 4 (2009) of the International Tobacco Control (ITC) Thailand Survey of adult smokers, which is part of the ITC Southeast Asia (ITC-SEA) Survey with a parallel survey in Malaysia. ITC Thailand is a nationally representative prospective cohort study designed to evaluate the psychosocial and behavioral impact of key national-level tobacco control policies. The first wave of data collection was in 2005 and the cohort was followed up in one or two year intervals and a small additional sample of smokers was obtained at each subsequent wave to replenish those lost to attrition. Follow-up surveys included respondents who had quit since the previous wave. The survey utilized a probability sampling design which involved a stratified multi-stage sample of individuals. The strata were Bangkok and two provinces from each of the four major regions. The primary sampling unit within each stratum was district. The second, third, and final stages of cluster sampling involved selection of subdistricts or communities, clusters of 300 households, and households, respectively. Finally, within each selected household, a maximum of two adult smokers were selected at random and invited to be interviewed face-to-face. All interviews were conducted in Thai. The combined eligibility and cooperation rate at Wave 1 in 2005 was 58.7%. Attrition from wave 1 to 2 was 22.1%, from wave 2 to 3 was 14.8%, and from wave 3 to 4 was 26%. For a more detailed description of the survey, refer to http://www.itcproject.org/.

### 2.2. Measurement

Two questionnaire items were used to measure smokers’ beliefs about “light” cigarettes: “Light cigarettes are less harmful than regular cigarettes” and “Light cigarettes are smoother on your throat and chest than regular cigarettes”. Respondents were asked to indicate their agreement with each statement on a five-point scale ranging from (1) “strongly disagree” to (5) “strongly agree”. These items have been used in previous research on beliefs about “light” cigarettes [25–28].

Baseline income and education were used as indicators of socioeconomic position. Annual household income was categorized into three groups of low (<36,000 baht), medium (>36,001 and <144,001 baht) and high (>144,000 baht). Similarly, education was categorized into three groups of low (no schooling and elementary), medium (lower and upper secondary), and high (diploma, bachelors degree, and higher). Only about 4% and 1% of respondents did not provide information on their level of income and education, respectively.

### 2.3. Statistical Analyses

Weighted data were used to compute mean of agreement and percentage that agreed or strongly agreed with beliefs about “light” cigarettes. Normal regression was employed to estimate the association of income, education, survey year, and control variables with beliefs about “light” cigarettes. In order to examine whether there was a socioeconomic difference in the effect of the policy, we assessed the interaction of income or education with survey year, all treated as categorical variables. Cases with missing values (n = 441) for any of the study variables were not included in the analysis. In order to take into account the correlated nature of the longitudinal data, we used generalized estimating equations (GEE) to compute parameter estimates [29], which were then used to calculate adjusted means for each outcome in each survey year. Our large sample size allowed us to assume an “unstructured” correlation structure in GEE. We used robust variance to compute *p*-values and confidence intervals [30]. Several time-varying control variables were considered in the multiple regression analyses: smoking status, number of cigarettes smoked per day, quit attempt in the past six months, use of “light” *versus* regular cigarettes, use of factory-made cigarettes *versus* roll-your-own (RYO) cigarettes *versus* a mix of both. Several time-invariant control variables were also considered: age, sex, residing in urban *versus* rural regions, residing in Bangkok *versus* other cities or towns, and wave of recruitment in the ITC Thailand. Only the variables that had a *p*-value smaller than 0.05 were kept in the multiple regression models. All analyses were conducted using Stata 10 SE [31].

**Figure 1.**
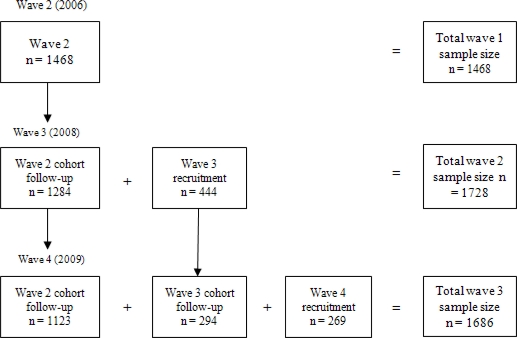
Sample size across waves 2, 3, and 4 of the International Tobacco Control (ITC) Thailand Survey.

The analysis included data from 2,352 respondents. Some were present in all three waves (*i.e.*, in years 2006, 2008, and 2009) of the current study, and others were present in one or two waves. Of all the respondents in the study, 882 were present in three waves, 766 in two waves, and 704 in only one wave (see Figure 1). A total of 4,882 person-wave observations were included in the regression models.

## 3. Results

The range of the age of the respondents was 18 to 86, with a mean of 43.95. About 92.51% of them were male and 44.15% lived in urban regions. Respondents with low, medium, and high income comprised 17.4, 51.0, and 31.6% of the sample, respectively. Those with low, medium, and high education comprised 64.8, 25.7, and 9.5% of the sample, respectively [32]. The sociodemographic characteristics of the sample were similar to those shown in a 2007 national survey, where 95.1% of smokers aged 15 years and older were male, and 66.1, 27.6 and 6.2% had low, medium and high level of education, respectively. In all survey years higher education and higher income were associated with a higher probability of using “light” *versus* regular cigarettes (*P* < 0.001). The percentage of smokers reporting using “light”, regular, or exclusively RYO was: 34.8, 24.8, and 40.3 in 2006; 18, 38.1, and 43.8 in 2008; and 22.8, 37, and 40.1 in 2009, respectively. Thus, the use of “light” cigarettes substantially decreased after the ban in 2008. There was no evidence that this change varied by income (*p* = 0.556) or education (*p* = 0.070).

Table 1 shows the mean level of agreement and percentage of respondents who agreed or strongly agreed with the two statements “Light cigarettes are less harmful than regular cigarettes” and “Light cigarettes are smoother than regular cigarettes”. The mean level of agreement for the “less harmful” belief was 3.06 (95% CI: 2.91–3.21), 2.63 (95% CI: 2.53–2.73), and 2.78 (95% CI: 2.69–2.86) in 2006, 2008, and 2009, respectively. The mean level of agreement for the “smoother” belief was 3.37 (95% CI: 3.30–3.45), 2.97 (95% CI: 2.84–3.10), and 2.89 (95% CI: 2.82–2.96), respectively. The pattern of change in percentage agreed or strongly agreed with the two statements mirrors those for the mean level of agreement.

**Figure 2.**
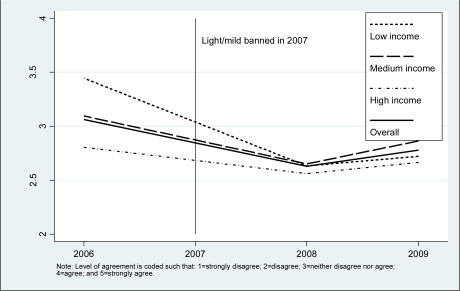
Mean of endorsement of ‘Light cigarettes are less harmful’ by income.

**Figure 3.**
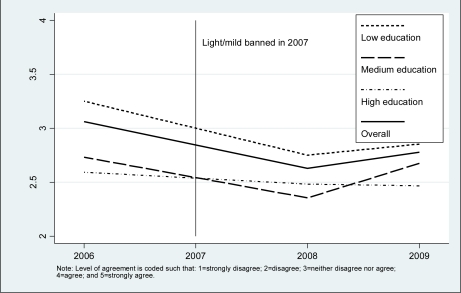
Mean of endorsement of ‘Light cigarettes are less harmful’ by education.

Figures 2 and 3 show the trend from 2006 to 2009 in the mean level of agreement with the “less harmful” statement by levels of income and education, respectively. In all survey years, low income and low education were associated with a stronger belief that “light” cigarettes are less harmful. The mean level of agreement in all groups declined after the introduction of the policy; however, there was a recovery in the mean of agreement in 2009, but it did not reach that of the 2006 baseline levels. Multiple regression analysis of the “less harmful” belief showed that there was an interaction between income and survey year (*p* = 0.006), and education and survey year (*p* = 0.029), which is consistent with the crude findings presented in Figures 2 and 3. Being a smoker *versus* a quitter (*p* < 0.001), older age (*p* < 0,001), and urban *versus* rural place of residence (*p* = 0.012) were associated with a higher mean of agreement with the “less harmful” belief. Table 2 shows the adjusted mean level of agreement by levels of income and education by year of survey. Overall, the mean declined from 3.07 in 2006 to 2.68 in 2008, after the introduction of the policy, but recovered slightly to 2.76 in 2009. The decline through the period of the study was much greater in the low income (from 3.34 in 2006 to 2.72 in 2009) than the high income group (from 2.84 in 2006 to 2.71 in 2009). Similarly, there was a much greater decline in the low education (from 3.21 in 2006 to 2.79 in 2009) than the high education group (from 2.58 in 2006 to 2.52 in 2009). This pattern of results indicated a decreasing trend in socioeconomic disparities in belief in harmfulness of “light” cigarettes.

Multiple regression analysis of the belief that “light” cigarettes are smoother provided no evidence for an effect of income (*p* = 0.884) or an interaction between income (*p* = 0.716) or education (*p* = 0.409) with survey year. Survey year was associated (*p* < 0.001) with the belief such that there was a steady decline in its adjusted (for the effect of age, smoking status, and education) mean from 3.37 in 2006, to 3.03 in 2008, after the introduction of the policy, and a recovery to 2.88 in 2009. Education also was associated with the belief (*p* < 0.001) such that its adjusted (for the effect of age, smoking status, and survey year) mean was 3.14, 2.97, and 2.96 for low, medium, and high education, respectively. Being a smoker *versus* a quitter (*p* < 0.001), use of “light” cigarettes (*p* = 0.001), and higher age (*p* = 0.006) were associated with a higher mean level of agreement.

## 4. Discussion

This was the first study to assess effects of the policy of banning the use of “light” descriptors on cigarette packs in Thailand. We found that, following the implementation of the policy, there was a decline in the belief that “light” cigarettes are less harmful than regular cigarettes, and that this decline was sharper among lower socioeconomic groups. While the belief that “light” cigarettes are smoother than regular cigarettes also declined, there was no evidence of a socioeconomic difference in the rate of decline. It is notable that postban levels of these beliefs remain quite high, higher than the levels reported in Western countries [27]. Thus, consistent to what has been found elsewhere [25], the policy, while effective to some extent, has not completely eliminated these incorrect beliefs and judgments.

The finding that lower socioeconomic smokers had a higher average level of endorsement of incorrect beliefs about “light” cigarettes were consistent with King and colleagues’ results which used the data from the first wave of the ITC Thailand and showed that lower education was associated with the belief that “light” cigarettes are healthier and easier to quit than regular cigarettes [26]. The findings were also consistent with the literature that lower socioeconomic position is associated with less knowledge of the harms of smoking [10,33–35].

The finding that, following the implementation of the policy, there was an overall decline in the level of endorsement of incorrect beliefs about “light” cigarettes was consistent with the results of our previous research in the United Kingdom (UK) [25]. After the removal of “light” and “mild” descriptors on cigarette packs in the UK in 2003, there was a decline in the level of endorsement of beliefs about health benefits of “light” cigarettes and the belief that “light” cigarettes are smoother. Following the initial decline, there was a rebound in the level of endorsement in the UK. Our data in the present analysis showed a similar rebound, albeit to a lesser extent, in the endorsement of the belief that “light” cigarettes are harmful. However, there was no rebound in the endorsement that “light” cigarettes are smoother; a steady decline continued throughout the study.

In order to further confirm the conclusion that the implementation of the policy in Thailand resulted in a decline in the belief that “light” cigarettes are less harmful and smoother, we conducted supplementary analyses using data from parallel surveys conducted by our group in Malaysia with identical questionnaire items. The weighted mean level of agreement with the belief that “light” cigarettes are less harmful in the sample from Malaysia remained relatively stable over time (2.58, 2.52, and 2.41, in 2006, 2008, and 2009, respectively), unlike the decline we reported above in Thailand. Similarly, the weighted mean level of agreement with the belief that “light” cigarettes are smoother in Malaysia did not show any evidence of a steady decline over time (3.16, 2.88, and 3.28, in 2006, 2008, and 2009, respectively), unlike the steady decline observed in Thailand. Using statistical modeling, we found overwhelming support for a difference in the pattern of change in each of the two beliefs over time between Malaysia and Thailand (p < 0.001). In may be argued that the decline in the level of misconceptions about “light” cigarettes is due to secular trend. However, this explanation can be largely ruled out because the 2005 level of agreement with the “less harmful” and “smoother” beliefs were very similar to the levels reported above for the year 2006, based on supplementary analyses not shown here of the ITC Thailand survey.

It should be noted that about half of the smokers in each wave of the study smoked RYO cigarettes and that our data showed that higher socioeconomic position is associated with a lower likelihood of using RYO. While the distinction between “light” and “regular” cigarettes are not made for RYO cigarettes, RYO smokers were aware of the “light” brand descriptors as very few of them indicated that they were unable to give an opinion on the statements that “light” cigarettes are less harmful or smoother.

A limitation of the study concerns the unmeasured confounders that might have played a role in the observed decline in the beliefs about “light” cigarettes examined in this article and how the effect of these confounders could have been different across socioeconomic position. For example, a difference in the change in the price of “light” *versus* regular cigarettes could affect consumption patterns and the rate of decline in the perception that “light” cigarettes are less harmful and smoother by socioeconomic position. Similarly, marketing strategies of the tobacco industry for “light” cigarettes and how these strategies might differ by the socioeconomic position of the industry’s target populations could have an impact on differences in the rate of decline in perceptions concerning “light” cigarettes.

Our findings suggest that information on packs may be a more important source of smoking-related beliefs in lower socioeconomic smokers. This could be because they are less likely to have information from other sources. As far as we know, there were no ancillary health education campaigns to reinforce the fact that “light” cigarettes are not less harmful. Given the socioeconomic disparities in smoking behavior and smoking-related beliefs [9–11], identifying or developing policies that can reduce these disparities should be a priority in tobacco control efforts. Removing misleading brand descriptors such as “light” on cigarette packs should be viewed not only as a means to address the problem of smokers’ incorrect beliefs about “light” cigarettes, but also as a factor that can potentially reduce smoking disparities in smoking-related misconceptions.

## Figures and Tables

**Table 1.** Crude mean level of agreement (95% CI) and percentage agree or strongly agree (95% CI) with “light cigarettes are less harmful” and “light cigarettes are smoother” by survey year.

	**Light cigarettes are less harmful**		**Light cigarettes are smoother**	
	**Mean level**	**% agree/strongly agree**	**Mean level**	**% agree/strongly agree**
	
**2006**	3.06 (2.91–3.21)	54.12 (47.36–60.89)	3.37 (3.30–3.45)	67.42 (63.39–71.45)
**2008**	2.63 (2.53–2.73)	36.03 (32.44–39.62)	2.97 (2.84–3.10)	48.09 (42.93–53.25)
**2009**	2.78 (2.69–2.86)	39.23 (35.77–42.69)	2.89 (2.82–2.96)	42.64 (39.62–45.66)

**Table 2.** Adjusted mean^a^ (95% CIs) level of agreement with “light cigarettes are less harmful”^b^.

	**Light cigarettes are less harmful**		
	**2006**	**2008**	**2009**

**Overall**	3.07 (3.01–3.13)	2.68 (2.62–2.73)	2.76 (2.70–2.82)

**Income**			

**Low**	3.34 (3.20–3.49)	2.71 (2.57–2.84)	2.72 (2.59–2.86)
**Medium**	3.10 (3.02–3.18)	2.68 (2.60–2.76)	2.81 (2.73–2.88)
**High**	2.84 (2.73–2.95)	2.65 (2.55–2.75)	2.71 (2.61–2.81)

**Education**			

**Low**	3.21 (3.14–3.29)	2.75 (2.68–2.82)	2.79 (2.72–2.86)
**Medium**	2.88 (2.76–3.01)	2.54 (2.42–2.65)	2.77 (2.66–2.89)
**High**	2.58 (2.39–2.77)	2.50 (2.31–2.68)	2.52 (2.33–2.70)

a Adjusted means are from a GEE model controlling for income, education, year, interaction of year and income, interaction of year and education, smoking status, age, and urban/rural.

b Level of agreement is coded such that: 1 = strongly disagree; 2 = disagree; 3 = neither disagree nor agree; 4 = agree; and 5 = strongly agree.
